# Exploring the divergence in perspectives on clinical trial operations in South Korea during the COVID-19 pandemic: a comparison of a trial site and sponsors

**DOI:** 10.3389/fmed.2024.1342184

**Published:** 2024-03-06

**Authors:** Young-Sang Kim, Anhye Kim, Yil-Seob Lee

**Affiliations:** ^1^CHA Global Clinical Trials Center, CHA Bundang Medical Center, CHA University, Seongnam, Republic of Korea; ^2^Department of Family Medicine, CHA Bundang Medical Center, CHA University, Seongnam, Republic of Korea; ^3^Department of Clinical Pharmacology & Therapeutics, CHA Bundang Medical Center, CHA University, Seongnam, Republic of Korea

**Keywords:** decentralized clinical trials, clinical trial methods, COVID-19 pandemic, sponsors, trial site

## Abstract

**Introduction:**

During the COVID-19 pandemic, novel clinical trial methods known as decentralized clinical trials (DCTs) were rapidly introduced. The attitude toward operating clinical trials and perspectives on DCTs may differ between clinical trial sites and sponsors. The impact of the COVID-19 pandemic on clinical trials was investigated for a society of sponsors and a trial site in South Korea.

**Methods:**

The current difficulties and future perspectives on clinical trials were assessed and compared between the site and sponsors.

**Results:**

Both the site and sponsors reported on their experiences with the challenges of conducting clinical trials during the pandemic era. While 64% of personnel from the site judged that the difficulties were solved by their own solutions, 67.6% of personnel from sponsors considered cooperation with trial sites as a key solution to overcome the difficulties. While half of the personnel from the site were skeptical of the changes in trial operation methods, the sponsors expected the institutionalization of DCT elements.

**Conclusion:**

In conclusion, with varying attitudes, sponsors and sites attempted to overcome the challenges of conducting clinical trials during the pandemic era. To conduct clinical trials effectively, both sponsors and sites must work closely together to find solutions with efficient communication. For the successful implementation of new tools such as DCTs, the government needs to solicit support from sponsors and sites and change regulations.

## 1 Introduction

In 2020, the COVID-19 outbreak compelled the governments of many countries to institute full or partial societal lockdowns (1). Korea also implemented various policies including social distancing and partial lockdown (2). The spread of COVID-19 imposed a burden on the healthcare system. The changes in the quarantine guidelines and people’s fear of infection minimized face-to-face contact, resulting in a reduction in the use of healthcare units (3).

While the COVID-19 pandemic accelerated the adoption of decentralized clinical trials (DCTs), the concept existed before the pandemic era. DCTs, also known as “direct-to-participant trials” or “virtual trials,” differ from traditional trials by leveraging telemedicine, mobile technology, and local healthcare providers (4, 5). This represents a paradigm shift in medical research, enabling remote and home-based participation, leading to increased accessibility for diverse populations, improved patient convenience, and potential cost-effectiveness (6). DCT leverages telemedicine, wearable devices, home nurses, and direct-to-patient shipments to gather data and provide care remotely, potentially increasing trial participation and efficiency (7). However, ethical considerations surrounding informed consent, data privacy, and participant vulnerability still present hurdles to widespread implementation (8).

The COVID-19 pandemic also had an impact on clinical trials around the world (9). Both trial sites and sponsors encountered challenges (10). Clinical trial participants tended to avoid face-to-face contact. Sponsors’ monitors had difficulty in visiting trial sites and inspecting them was also challenging in some ways. Changes in the clinical trial environment hastened the transition to new approaches to clinical trial operation (11). Although these new approaches were interested in the era of the COVID-19 pandemic, in some countries, these methods may be difficult to apply due to regulatory hurdles. In addition, the viewpoints of the new methods may differ between trial sites and sponsors. Our study explored divergence in perspectives on conducting clinical trials during the COVID-19 pandemic, particularly regarding readiness for DCTs, between a trial site and sponsors in Korea.

## 2 Materials and methods

### 2.1 Definitions of the site and the sponsor

In clinical trials, two key stakeholders have distinct roles and responsibilities. A clinical trial site is defined a location where participants are enrolled and undergo the trial procedures. Research sponsors are the individual, company, or organization that initiates, funds, and manages the clinical trial. Contract research organizations (CROs) provide outsourced support to sponsors in various aspects of clinical trials. CROs are often deeply integrated with pharmaceutical companies and have the capability to directly connect both research sponsors and sites. This close relationship brings their role closer to that of a sponsor in practice. Therefore, in this study, CROs are regarded as sponsor sides.

### 2.2 Methods

The current study was conducted based on two surveys for Korea Society for Clinical Development (KSCD) and a survey for a domestic trial site. KSCD consists of pharmaceutical companies, CROs, hospitals, and related organizations for clinical development. The surveys of KSCD were conducted for the member companies to investigate the sponsors’ operation of clinical trials under the pandemic. KSCD surveys were sent to the member companies via email, and the received responses were input and analyzed. The survey of CHA Bundang Medical Center examined the investigators’ opinion for influence of COVID-19 on clinical trials in a trial site. This survey was sent to site investigators using Google Forms, and the received responses were analyzed. The original questionnaires of the surveys and English-translated versions are provided in Supplementary material.

### 2.3 Surveys of KSCD

In July 2020, KSCD surveyed the impact of the COVID-19 pandemic on clinical trials in South Korea (Survey S1). The questionnaire covers the difficulties caused by COVID-19, the solutions to the difficulties, and the prospects after the pandemic to share experiences to develop drugs during the pandemic. Only one representative from each member company is allowed to respond to the questionnaire. A total of 42 companies voluntarily responded to the survey (Table 1). Personnel from domestic and multinational pharmaceutical companies (*N* = 14 and 16, respectively) and domestic and multinational CROs (*N* = 7 and 5, respectively) responded. Half of the respondents (*N* = 22) were operation heads, managers, or directors.

**Table 1:** The general characteristics.

Site investigators (*N* = 33)		Sponsors (year 2020; *N* = 42)	
Position		Characteristic	
Professor	14 (42.4%)	Domestic pharmaceutical company	14 (33.3%)
Associate professor	13 (39.4%)	Multinational pharmaceutical company	16 (38.1%)
Assistant professor	4 (12.1%)	Domestic CRO	7 (16.7%)
Clinical research coordinator	2 (6.1%)	Multinational CRO	5 (11.9%)
Number of ongoing trials		Position	
20 or more	2 (6.1%)	Project manager	8 (19.0%)
10–20	1 (3.0%)	CRA/PM line manager	6 (14.3%)
5–10	12 (36.4%)	Operation unit head/manager/director	22 (52.4%)
1–5	15 (45.5%)	General manager	6 (14.3%)
0	3 (9.1%)		
The phase of ongoing trials (*N* = 30)		Sponsors (year 2021; *N* = 36)	
Phase 1	13 (43.3%)	Characteristic	
Phase 2	16 (53.3%)	Domestic pharmaceutical company	7 (19.4%)
Phase 3	21 (70.0%)	Multinational pharmaceutical company	17 (47.2%)
Phase 4	10 (33.3%)	Domestic CRO	5 (13.9%)
PMS	11 (36.7%)	Multinational CRO	7 (19.4%)

CRO, a contract research organization; CRA, a contract research associate; PMS, post-marketing surveillance.

In 2021, KSCD surveyed post-COVID-19 DCT elements. The questionnaire consists of opinions on the expected DCT elements after COVID-19, whether any of these elements have been attempted, and whether there were any challenges encountered during the process. A total of 36 businesses responded (Table 1). The survey included questions about the current state of DCT elements and their prospects.

### 2.4 Survey of CHA Bundang Medical Center

In August 2021, a survey on the impact of the COVID-19 pandemic on clinical trials was conducted at CHA Bundang Medical Center in South Korea. A total of 33 personnel participated in this survey (Table 1). This survey also consists of the questionnaires of difficulties due to COVID-19, the solution for the difficulties, and the perspectives after the pandemic including preparedness for DCTs. Most responders were professors as site investigators.

### 2.5 Ethics statement

While the original surveys were conducted independently with their own consent procedures, this secondary analysis did not require additional informed consent from participants as it used anonymized data already collected and approved by the corresponding IRBs. The ethics approval for the secondary analysis of this study was waived by the Institutional Review Board of CHA Bundang Medical Center (2022-03-041).

## 3 Results

### 3.1 Challenges in conducting clinical trials due to COVID-19

In the pandemic era, personnel from both the site and sponsors face several challenges when conducting clinical trials (Table 2). Half of the respondents from both sides (43.3% from the site and 61.9% from the sponsors) thought that the clinical trials had been postponed, canceled, or discontinued. The site’s most common response was that it was difficult to enroll subjects (70.0%). Similarly, the most common response from sponsors was a delay in the schedule due to enrollment difficulties (85.7%).

**Table 2:** Challenges in conducting clinical trials during COVID-19.

Site investigators (*N* = 30)	
Difficulties enrolling subjects	21 (70.0%)
Difficulties meeting with sponsor staff	16 (53.3%)
Trial postponement, cancelation, or discontinuation	13 (43.3%)
Difficulties adhering to the protocol	11 (36.7%)
Participant withdrawal of consent	1 (3.3%)
Sponsors (*N* = 42)	
Delay in schedule due to enrollment difficulties	36 (85.7%)
Trial postponement, cancelation, or discontinuation	26 (61.9%)
Difficulties adhering to the protocol	23 (54.8%)
Difficulties managing research personnel	19 (45.2%)
Increase in trial budget	11 (26.2%)
Low data quality	7 (16.7%)
Business management difficulties (e.g., contract cancelation)	6 (14.3%)
Other responses	5 (11.9%)
None	2 (4.8%)

### 3.2 Overcoming challenges in conducting clinical trials

**Figure 1:**
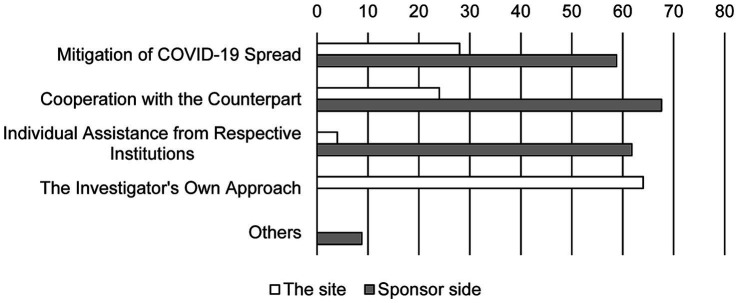
Experience in resolving problems. Bars show the response rate. Duplicate responses are allowed (*N* = 25 in the site and *N* = 34 in sponsor side).

Twenty-five from the site and 34 from sponsors responded with their experiences of problem-solving (Figure 1). Most of the personnel from the site (64%) believed that the difficulties were resolved by their own solution. Contrarily, sponsors responded their varied experiences to be beneficial in overcoming challenges. Although many personnel from sponsors (67.6%) considered cooperation from the sites to be helpful, relatively few investigators (24%) felt that cooperation from the sponsors was supportive. A significant number of personnel from sponsors (61.8%) responded that companies provided revised guidelines to solve the challenges in conducting clinical trials.

**Figure 2:**
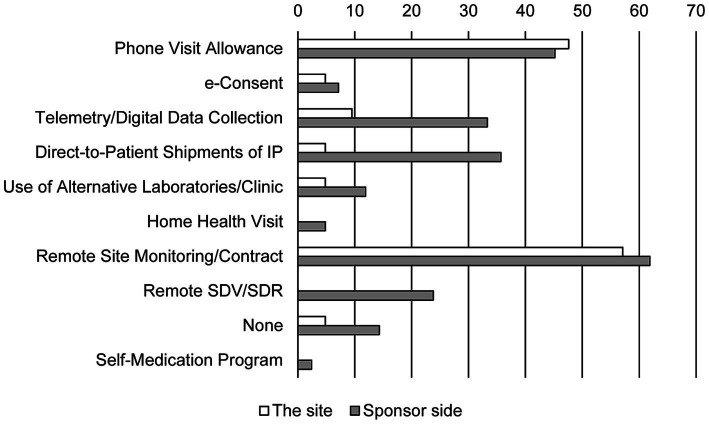
DCT methods attempted for resolving problems. Bars show the response rate. Duplicate responses are allowed (*N* = 25 in the site and *N* = 34 in sponsor side). IP, investigational product; SDV, source data verification; SDR, source data review.

Sponsors tended to try to incorporate different types of DCT elements (Figure 2). While phone visits and remote monitoring were widely used, digital data collection including wearables, direct-to-patient shipments were also utilized. However, alternative laboratories and home health visits were not commonly incorporated. In contrast, site investigators were far from DCT elements when attempting to solve the problems. The investigators’ most common method of contact with the subjects (47.6%) was simply by phone.

### 3.3 The perspectives on the changes in the operation of clinical trials after the pandemic

**Figure 3:**
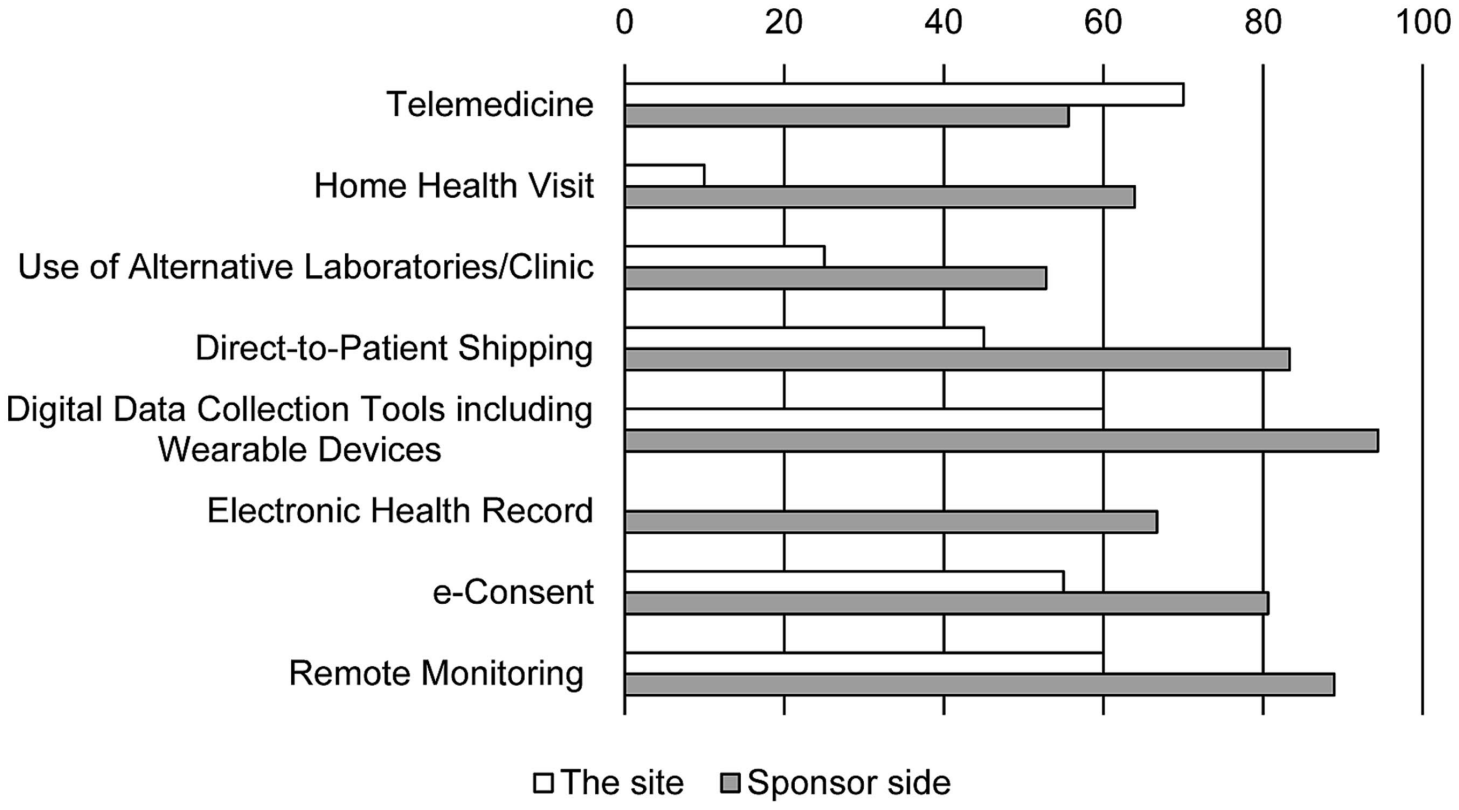
The perspectives on post-pandemic changes in DCT elements. Bars show the response rate. Duplicate responses are allowed (*N* = 20 in the site and *N* = 36 in sponsor side). Item of electronic health records was absent in the survey for the site.

**Figure 4:**
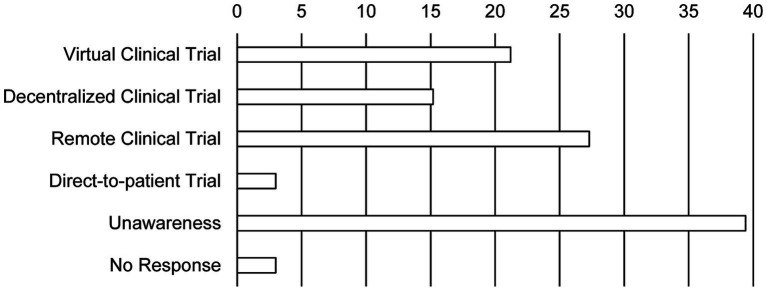
The investigators’ awareness of new clinical trial terminology. Bars show the response rate. Duplicate responses are allowed (*N* = 25 in the site).

The sponsors hoped that DCT elements are needed to be institutionalized soon and looked forward to regulatory changes that would accept DCT tools (Figure 3). In contrast, half of the personnel at the site never expected the changes in the methods of the clinical trial (45.5%). While the personnel at the site ranked direct contact with the aid of telemedicine tools such as remote access, e-consents, and remote monitoring as new methods, methods that use home care nurses and alternative clinics instead of the real sites were not favored. Furthermore, 39.4% of the personnel at the site were not aware of terminologies indicating new trial methods such as virtual trial, remote trial, direct-to-patient trial, and particularly DCT (Figure 4).

## 4 Discussion

In the pandemic era of COVID-19, both the trial sites and sponsors have faced many challenges in conducting clinical trials. The most important tasks in resolving these issues are preventing the spread of the pandemic and communicating effectively with trial sites and sponsors. While the site investigators favored indirect contact using telephone and email, the sponsors desired to introduce various tools of DCTs.

The investigators at the trial site strongly believed that the difficulties could be overcome by themselves despite the pandemic environment. In addition, half of the personnel at the site not only were unfamiliar with the terminologies of novel methods in clinical trials but also had skeptical insights into them. They advocated for a cautious approach to changes in clinical trial methods. With sponsors’ support, site investigators must take action against the changes (12). Our study showed a gap between site investigators and sponsors. Sponsors are more prepared to use DCT elements than site investigators. As site investigators do not recognize the benefits of DCT in terms of patients’ perspectives, both parties must communicate more efficiently with one another (13). As sponsors are required to train clinical research associates (CRAs), CRAs need to play more roles in communicating with site personnel (14). Each site needs to prepare a new standard operating procedure according to the government guidelines and to train affiliated personnel.

As known well, the COVID-19 pandemic disrupted many clinical trials that were potentially bringing new therapeutics to market (15). As a result of this crisis, the US Food and Drug Administration, alongside its international counterparts, has developed guidance to protect both research participants and trials by advocating for remote data collection supplemented by telemedicine (16). Regulatory authorities must set the direction for resolving these issues by developing guidelines. According to global data 2021, digitalization of preclinical and clinical trials was viewed positively by 39% of healthcare and pharmaceutical professionals in North America, 39% in Europe, and 28% in the Asia-Pacific region (17). In our study, clinical trial sponsors believed that DCT elements, such as digitalization, were required for successful clinical trials even after the pandemic era. However, some DCT elements cannot be used in some countries, including South Korea, due to regulatory hurdles (18, 19). Although the aggressive adoption of DCT services and technology interventions is expected after the pandemic in UK, US, and France, Japan is facing its own challenges while trying to adopt this trend, similar to South Korea (20, 21). Hence, regulatory authorities need to identify hurdles with sponsors and sites and solve them. In addition, trial sites also need to support remote monitoring performed by sponsors.

This study has several limitations. First, contrary to the sponsors, only one trial site was considered for the survey. Although overestimation of our result is a caution, it is advisable to see a viewpoint of the personnel working at the site. Second, one of the surveys for the sponsors was conducted the previous year; due to the interval between the surveys, the pandemic status, policy response, and social attitude may be different from the two points. However, even in this advanced year, it is interesting to note that many site investigators remain unaware of new approaches to conducting clinical trials. Third, there was confusion regarding several DCT components. The survey asked about home health visits instead of mobile nursing systems. Additionally, digital data collection encompasses both wearable devices and ePRO/eCOA, not just one or the other. Fourth, the subjects who participated in clinical trials were not surveyed. Changes in trial methods have a direct impact on the subjects, and the changes must be geared toward the subjects’ benefit and convenience. Lastly, the actual implementation of DCT items during the COVID-19 period was not sufficiently described. It is necessary to investigate whether it will be conducted in an appropriate manner following the standard protocol.

In conclusion, despite many common challenges in conducting clinical trials during the pandemic era, sponsors and sites were trying to overcome them with different attitudes. The COVID-19 pandemic has accelerated the implementation of DCT solutions, and DCT will be used in clinical trials more and more in the future, with benefits for sponsors and sites. Furthermore, decentralized solutions improve the patient experience. Both sponsors and sites need to have close communication and find solutions when they face challenges like COVID-19 in conducting clinical trials. In addition, sponsors and sites need to share feasible solutions and coordinate to train the personnel involved in the clinical trial. The government needs to gather opinions from sponsors and sites and change regulations for the successful implementation of new tools like DCT.

## Data availability statement

The original contributions presented in the study are included in the article/Supplementary material, further inquiries can be directed to the corresponding author.

## Author contributions

Y-SK: Data curation, Formal analysis, Investigation, Software, Visualization, Writing – original draft, Writing – review & editing. AK: Methodology, Validation, Writing – review & editing. Y-SL: Conceptualization, Investigation, Methodology, Validation, Writing – review & editing.

## Supplementary material

The Supplementary material for this article can be found online at:

https://www.frontiersin.org/articles/10.3389/fmed.2024.1342184/full#supplementary-material
