# Supplementary material for: Exploring the divergence in perspectives on clinical trial operations in South Korea during the COVID-19 pandemic: a comparison of a trial site and sponsors
Source: Front Med (Lausanne). 2024 Mar 6;11:1342184. doi: 10.3389/fmed.2024.1342184 (PMC10950924; doi:10.3389/fmed.2024.1342184)
Supplement: Supplementary file 1 [file Data_Sheet_1.PDF]

KSCD Survey in July 2020

Name of company:

Your Name:

1. What is the characteristic of your company?

- ☐ Domestic pharmaceutical company
- ☐ Multinational pharmaceutical company
- ☐ Domestic CRO
- ☐ Multinational CRO

2. What is your role in your field?

- ☐ Project manager
- ☐ CRA/PM line manager
- ☐ Operation unit head/manager/director
- ☐ General manager

3. What is your difficulties in your field or company due to COVID-19 pandemic?

① Postponement/canceler/discontinuation of new trials

② Increment in budget for trials

③ Difficulties to manage research manpower

④ Difficulties in business management (e.g., rescission of contracts)

⑤ Delay of schedule due to enrollment difficulty

⑥ Difficulties to obey the protocol

⑦ Low reliability on data quality

⑧ Other responses

4. What is the concrete difficulties if you chose 5–7?

① Difficulties to enroll subjects

② Happening of deviations

- Deviations of visit window
- Missing visit
- Withdrawal
- Lost follow up
- Temporary treatment discontinuation
- Premature treatment discontinuation
- Missing laboratory or other tests

③ Difficulties in monitoring

④ Site close down

⑤ Site staff unavailability

⑥ Other responses

5. Did your difficulties solved?

- ☐ Solved
- ☐ Partly solved
- ☐ Not solved

5-1. How did you solve the problem if you chose 1–2 following above question #5?

- ☐ Mitigation of COVID-19 spread
- ☐ Cooperation of sites
- ☐ Provision of guidelines by the company
- ☐ Others

5-2. What is unresolved if you chose 1–2 following above question #5?

① Difficulties to enroll subjects

② Happening of deviations

- Deviations of visit window
- Missing visit
- Withdrawal
- Lost follow up
- Temporary treatment discontinuation
- Premature treatment discontinuation
- Missing laboratory or other tests

③ Difficulties in monitoring

④ Site close down

⑤ Site staff unavailability

⑥ Other responses

6. What is your solutions to overcome difficulties?

① Allowance of phone visit

② Introduction of e-consent

③ Telemetry/digital data collection

④ Direct-to-patient shipments of IP

⑤ Central lab

⑥ Home health nurse system

⑦ Remote site monitoring

⑧ Remote SDV/SDR

⑨ Other responses

7. Please describe your discussion point on the difficulties amid COVID-19 pandemic.

8. Please provide your opinion for government to reduce the difficulties in conducting trials and to mitigate the regulations.

KSCD Survey in August 2021

Decentralized Clinical Trial Survey

Name of company:

Your Name:

Choose the characteristic of your company

- ☐ Domestic pharmaceutical company
- ☐ Multinational pharmaceutical company
- ☐ Domestic CRO
- ☐ Multinational CRO

1. What is the predicted trial methods after COVID-19 pandemic?

- ■ Telemedicine
- ■ Home Health Visits
- ■ Local Lab Use
- ■ Direct to Patient (DtP) Shipping
- ■ Digital Data Collection Tools
- ■ Electronic Health Record & Electronic Data Capture
- ■ Electronic Informed Consent
- ■ Remote Monitoring and Sponsor Visits to Study Sites

2. What was tried among the above list in our country?

Select one for each item from the following list

0. Never tried, or having no plan
1. Tried, but not proceeded
2. Now trying or having plans
3. Now conducting
4. Not tried, but waiting for a better environment

- Telemedicine
- Home Health Visits
- Local Lab Use
- Direct to Patient (DtP) Shipping
- Digital Data Collection Tools
- Electronic Health Record & Electronic Data Capture
- Electronic Informed Consent
- Remote Monitoring and Sponsor Visits to Study Sites

3. What is difficult when trying the following item in our country?

Select one for each item from the following list

0. Not difficult
1. Absence of the law or act
2. Deficiency in SOP, system, or personnel in trial sites
3. Difficulties in agreement with site investigators
4. Other opinion

- Telemedicine
- Home Health Visits
- Local Lab Use
- Direct to Patient (DtP) Shipping
- Digital Data Collection Tools
- Electronic Health Record & Electronic Data Capture
- Electronic Informed Consent
- Remote Monitoring and Sponsor Visits to Study Sites

4. If you chose "4. Other opinion" in any item following the question #3, please describe in detail.

5. Please provide an appropriate Korean term for Decentralized Clinical Trial.

6. Please provide a suggestion to improve the environment to conduct trials in Korea.

Survey in CHA Bundang Medical Center in August 2021

Survey on the influence of COVID-19 on clinical trials

1. Department:

2. Name:

3. Position

   A. Professor

   B. Associate professor

   C. Assistant professor

   D. Clinical research coordinator

4. Have you participated in clinical trials between January 2020 and July 2021? How many trials do you participate?

   A. 20 or more

   B. 10–20

   C. 5–10

   D. 1–5

   E. 0

5. If you participate in trials, which phase is the trial?

   A. Phase 1

   B. Phase 2

   C. Phase 3

   D. Phase 4

   E. PMS

6. What is your difficulties due to COVID-19?

   A. Postponement/canceler/discontinuation of trials

   B. Difficulties to enroll subjects

   C. Withdrawal of consents

D. Difficulties to obey the protocol
E. Difficulties to meet sponsor staff

7. What have you experienced among the difficulties to obey the protocol?

A. Deviations of visit time
B. Failure in subjects' visit
C. Failure in follow-up contacts with subjects
D. Temporary treatment discontinuation
E. Permanent treatment discontinuation
F. Difficulties in assessment related to clinical trials

8. How have the difficulties been solved?

A. Mitigation of COVID-19 spread
B. Cooperation of sponsors
C. Support from site hospital
D. Investigator's solution

9. If you chose C and/or D following question #8, please describe your know-how concretely.

10. Please choose the methods you have done to overcome the difficulties?

A. Phone contact instead of direct visit
B. Using e-consent
C. Remote data collection
D. Use of local clinic
E. Shipment of IP by parcel service
F. Visit by home health nurses
G. Contact to sponsor personnel using phone or email

11. Do you think the operation system in clinical trials will be changed after the end of COVID-19 pandemic?

A. No changes, return to the past

B. New methods including telemedicine and intact trial

12. If you choose “new methods”, what would be attempted among the lists?

A. e-Consent

B. Remote practice

C. Direct-to-patient shipments of IP

D. Remote assessment

E. Visit by home health nurses

F. Use of local clinic

G. Remote site monitoring

13. Which terms indicating new trial methods have you heard of?

A. Virtual clinical trial

B. Decentralized clinical trial

C. Remote clinical trial

D. Direct-to-patient trial

14. What kinds of suggestions or help are required for the attempt of the new clinical trial methods? Please describe your idea concretely to each of health authority, sponsors, and hospital, respectively.

안녕하십니까.

한국 임상개발연구회(이후 임연회)에서는 회원사를 대상으로 COVID-19 의 영향 및 이로 인한 어려움을 극복하는 데 도움이 될 만한 부분을 파악하고자 다음의 설문 조사를 진행하고자 합니다.

본 설문에서 수합된 결과는 회원사께 공유되며, 식약처를 포함한 관련 단체와의 논의 시 활용될 예정입니다.

회원사 내에서 본 설문 내용에 대해 잘 알고 계신 한 분께서 작성해주실 수 있도록 공유해 주시면 감사하겠습니다.

회사명:

작성자 성명:

1. 해당하는 회원사의 종류를 선택하여 주십시오.
    - ○ 국내 제약사
    - ○ 다국적 제약사
    - ○ 국내 CRO
    - ○ 다국적 CRO
    - ○ 기타:

2. 귀하의 업무와 가장 근접한 업무 영역을 선택하여 주십시오.
    - ○ CRA
    - ○ Project Manager
    - ○ CRA/PM Line manager
    - ○ Operation Unit Head / Manager / Director
    - ○ General Manager

3. COVID-19 로 인해 회원사께서 <u>겪었거나, 겪고 있는 어려움</u>이 있다면 선택해 주시기 바랍니다 (우선 순위 선택, 다수 선택 가능)
    1) 신규 임상연구 계획의 연기, 취소 또는 중단
    2) 임상 연구 비용의 증가
    3) 임상 연구 인력의 활용의 어려움 (연구 지연, 취소 및 중단으로 인해)
    4) 사업 운영의 어려움 (사업 계약의 취소 등으로 인해)
    5) 대상자 등록의 어려움으로 인해 연구 일정의 지연
        - 약 6 개월 이내의 지연
        - 약 6 개월 - 12 개월 지연
        - 약 12 개월 이상의 지연
    6) 임상 연구 계획서를 준수하도록 하기 위한 노력 (임상약의 배송 등)
    7) 임상 연구의 Data Quality 문제 (결과의 신뢰성)
    8) 기타:

4. 위 질문에서 5), 6), 7)을 선택한 경우, 구체적인 어려움은 어떤 것이었는지 선택해 주시기 바랍니다. (우선 순위 선택, 다수 선택 가능)

1) 대상자 등록의 어려움
2) 여러 Deviation 의 발생
    - 대상자의 방문일 (Visit window) 위반
    - 대상자의 방문 불가능 (Missing Visit)
    - 대상자의 동의 철회 (Withdrawal)
    - 대상자의 추적조사 불가능 (Lost Follow Up)
    - 일시적 임상약 투여 중단 (Temporary Treatment Discontinuation)
    - 영구적 임상약 투여 중단 (Premature Treatment Discontinuation)
    - 각종 검사 불가능 (missing laboratory or other test)
3) 모니터링 불가능 또는 어려움
4) 기관의 폐쇄 (Site Close Down)
5) 기관 Staff Unavailability
6) 기타:

5. COVID-19 로 인해 회원사께서 겪으셨거나 겪고 있는 어려움이 해결되었습니까?

1) 해결
2) 일부 해결
3) 전혀 해결되지 않음

5-1. 위 질문 5 에서 1) 또는 2) 로 선택한 경우, 어떻게 해결되었습니까?

1) COVID-19 완화
2) 참여 기관의 협조
3) 회사내 방안 마련 및 가이드라인 제공
4) 기타:

5-2. 위 질문 5 에서 2) 또는 3)으로 선택한 경우, 현재 어떤 어려움이 남아 있습니까? (4 번에서 선택한 내용 중에 진행중인 어려움을 다시 선택)

1) 대상자 등록의 어려움
2) 여러 Deviation 의 발생
    - 대상자의 방문일 (Visit window) 위반

- 대상자의 방문 불가능 (Missing Visit)
- 대상자의 동의 철회 (Withdrawal)
- 대상자의 추적조사 불가능 (Lost Follow Up)
- 일시적 임상약 투여 중단 (Temporary Treatment Discontinuation)
- 영구적 임상약 투여 중단 (Premature Treatment Discontinuation)
- 각종 검사 불가능 (missing laboratory or other test)

3) 모니터링 불가능 또는 어려움
4) 기관의 폐쇄 (Site Close Down)
5) 기관 Staff Unavailability
6) 기타:

6. 기타: 다음의 항목 중 회원사에서, COVID-19 로 인한 어려움을 극복하기 위해 활용한 것이 있었습니까? (다수 선택 가능, 기존에 사용하였더라도 COVID-19 로 인한 어려움을 극복하는데 도움이 되었다면 선택 가능)
    1) 전화방문 (Phone Visit) 허용
    2) 전자동의서 (Electronic Informed Consent) 도입
    3) 원격데이터 수집 (Telemetry/Digital Data Collection tools, 예: e-CRF, e-Diary, e-PRO 등)
    4) 대상자에게 직접 임상약을 배송 (Direct To Patient Shipment of Trial Product)
    5) 기존 실험/검사실 대신 이용 가능한 실험/검사실의 이용 (Central <-> Local Laboratory)
    6) 가정 간호시스템 도입 (Home/Mobile Nursing)
    7) Remote/Off-Site 모니터링 방문 (Selection, Initiation, Monitoring, Close Out Visit 을 포함)
    8) Remote SDV/SDR
    9) 기타:

7. COVID-19 로 인해 겪고 계신 어려움 중에 임연회 또는 회원사들로부터 도움이 필요하거나 공유되었으면 하는 정보가 있다면 의견 부탁드립니다.

8. COVID-19 로 인해 겪고 계신 어려움 및 이후의 유사한 상황에 대처하기 위해 식약처 또는 임상시험 관련 규제기관/단체에 제안하고 싶은 부분 (제도적인 개선)이 있다면 의견 부탁드립니다.

Decentralized Clinical Trial Survey

안녕하십니까.

한국 임상개발연구회(이하 임연회)에서는 회원사를 대상으로 COVID-19 팬데믹과 함께 전세계적으로 가속화되고있는 Decentralized Clinical Trial(이하 DCT) 도입과 관련한 국내 현황을 파악하고, 국내 DCT 환경 조성에 있어 어떠한 DCT 요소들이 시급하게 논의되어야 할지를 알아보고자 다음의 설문 조사를 진행하고자 합니다.

본 설문에서 수합된 결과는 회원사께 공유되며, 식약처를 포함한 관련 단체와의 논의 시 활용될 예정입니다. 결과 공개시 회사명/작성자 성명은 공개되지 않습니다.

회원사 내에서 본 설문 내용에 대해 잘 알고 계신 한 분께서 작성해주실 수 있도록 공유해 주시면 감사하겠습니다.

회사명:______________________________

작성자 성명:__________________________

해당하는 회원사의 종류를 골라주세요.

□국내 제약사
□다국적 제약사
□국내 CRO
□다국적 CRO
□기타: __________________________

1. 회사의 전략상 Covid-19 팬데믹 이후 사용이 확대될 것으로 예상되는 시도를 모두 표시해주십시오. (외자사의 경우, 본사 차원에서 답변바랍니다.)

☐ Telemedicine (원격진료)
☐ Home Health Visits (가정방문간호)
☐ Local Lab Use (지역병원에서 검사)
☐ Direct to Patient (DtP) Shipping (기관에서 환자집으로 직접 약 배송)
☐ Digital Data Collection Tools (전자정보수집장치/기기, ePRO, eCOA, wearables 등)
☐ Electronic Health Record & Electronic Data Capture (전자의무기록지 & 전자자료 수집, EMR 자료의 CRF로의 자동전송 등)
☐ Electronic Informed Consent (전자동의서, eConsent)

☐ Remote Monitoring and Sponsor Visits to Study Sites (원격모니터링/의뢰자의 원격방문, Remote SDV/SDR)

2. 국내에서는 어떤 것들이 시도/진행된 적이 있나요?

	0-시도한적 없고, 향후계획없음.	1-시도하였으나 진행되지 못함.	2-시도 중이거나 향후 계획있음.	3-진행 중	4-시도한적은 없지만, 가능한 환경이 되면 진행하고자 함.
Telemedicine (원격진료)	☐	☐	☐	☐	☐
Home Health Visits (가정방문간호)	☐	☐	☐	☐	☐
Local Lab Use (지역병원에서 검사)	☐	☐	☐	☐	☐
Direct to Patient (DtP) Shipping (기관에서 환자집으로 직접 약 배송)	☐	☐	☐	☐	☐
Digital Data Collection Tools (전자정보수집장치/기기, ePRO, eCOA, wearables 등)	☐	☐	☐	☐	☐
Electronic Health Record & Electronic Data Capture (전자의무기록지 & 전자자료 수집, EMR자료의 CRF로의 자동 전송 등)	☐	☐	☐	☐	☐
Electronic Informed Consent	☐	☐	☐	☐	☐

(전자동의서, eConsent)					
Remote Monitoring and Sponsor Visits to Study Sites (원격 모니터링/의뢰자의 원격방문, Remote SDV/SDR)	☐	☐	☐	☐	☐

*이 질문에서는 행마다 하나의 응답을 작성해야 합니다.

3. 국내에서 시도/진행시 어려움이 큰 이유는 무엇인가요?

	0-어려움 없이 시도/진행	1-국내규정 미비	2-국내규정은 있으나 연구기관에서의 SOP/시스템/인력 부족	3-국내규정 및 연구 기관의 SOP는 있으나, 연구진과의 합의에 어려움	4-기타
Telemedicine (원격진료)	☐	☐	☐	☐	☐
Home Health Visits (가정방문간호)	☐	☐	☐	☐	☐
Local Lab Use (지역병원에서 검사)	☐	☐	☐	☐	☐
Direct to Patient (DtP) Shipping (기관에서 환자집으로 직접 약 배송)	☐	☐	☐	☐	☐
Digital Data Collection Tools (전자정보수집장치/기기, ePRO, eCOA, wearables	☐	☐	☐	☐	☐

등)					
Electronic Health Record & Electronic Data Capture (전자의무기록지 & 전자자료 수집, EMR자료의 CRF로의 자동 전송 등)	☐	☐	☐	☐	☐
Electronic Informed Consent (전자동의서, eConsent)	☐	☐	☐	☐	☐
Remote Monitoring and Sponsor Visits to Study Sites (원격 모니터링/의뢰자의 원격방문, Remote SDV/SDR)	☐	☐	☐	☐	☐

*이 질문에서는 행마다 하나의 응답을 작성해야 합니다.

4. 3 번 질문에서 "기타"를 선택한 경우, 자세한 설명 부탁드립니다.

5. 현재까지 탈중심화 임상, 비대면 임상 등으로 DCT 가 국내 소개된 바 있으나 아직 DCT 에 대한 국문용어가 정해지지는 않았습니다. Decentralized Clinical Trial (DCT)에 대한 적합한 국문용어 제안과 그 이유를 간단히 써주시면 감사하겠습니다.

6. 국내 임상환경 (DCT 환경 조성포함)을 개선하기위한 제안사항

코로나19가 임상시험에 끼친 영향에 대한 설문조사

코로나19로 인하여 임상시험의 진행에 어려움을 겪는 연구자들이 많이 있는 것 같습니다. 어떤 부분에서 어려움이 있는 지, 그 어려움을 임상 현장에서 어떻게 해결하시고 계시는지 그리고 코로나19가 종식되면 임상시험 환경에 어떤 변화를 예측하시는 지 등을 파악하고자 설문을 진행하고자 합니다. 아래 설문에 답변해 주시면 감사하겠습니다. 본 설문에서 취합된 결과는 추후 교수님들에게 공유하도록 하겠습니다. 교수님이 직접 답변이 어려우시면 담당 CRC를 통해서 답변 주셔도 될 것 같습니다. 감사합니다.

1. 소속과: ____________
2. 작성자 성명: ________________
3. 직위?
   A. 교수
   B. 부교수
   C. 조교수
   D. 임상강사
4. 코로나19가 발생한 2020년 1월부터 2021년 7월말까지 임상시험에 참여하신 경험이 있으신지요? 있으시다면 총 몇 건 임상시험에 참여하고 계신지요?
   A. 20건 이상
   B. 10~20건
   C. 5~10건
   D. 1~5건
   E. 0건
5. 참여하셨다면 어느 단계 임상시험에 참여하셨나요 (복수 답변 가능) ?
   A. 1상 시험
   B. 2상 시험
   C. 3상 시험

D. 4상 시험

E. PMS

6. 코로나19로 인하여 발생한 어려움은 어떤 것이었는지요 (복수 답변 가능) ?

A. 임상시험 계획의 연기, 취소 또는 중단

B. 대상자 등록의 어려움

C. 대상자 동의 철회

D. 임상시험 계획서 준수의 어려움

E. 스폰서 직원과의 미팅의 어려움

7. 위에 답변하신 임상시험 계획서 준수의 어려움 중 어떤 것들을 경험하셨나요 (복수 답변 가능)?

A. 피험자 방문일 위반으로 인한 계획서 준수 어려움

B. 대상자 방문 불가능으로 인한 계획서 준수 어려움

C. 대상자 추적 조사 불가능으로 인한 계획서 준수 어려움

D. 임상시험용 의약품 일시적 투약 중단으로 인한 계획서 준수 어려움

E. 임상시험용의약품 영구적 투약 중단으로 인한 계획서 준수 어려움

F. 임상시험관련 검사 진행의 어려움으로 인한 계획서 준수 어려움

8. 어려움이 해결된 경우, 어떻게 해결되었나요 (복수 답변 가능)?

A. 코로나19 상황 완화

B. 스폰서가 방안을 마련해 줌

C. 병원에서 해결책을 마련해 줌

D. 연구자가 스스로 해결 함

9. 8번 질문에서 C와 D에 답변하신 경우, 구체적으로 어떤 해결책을 사용하셨는지 기재 부탁 드립니다.

10. 어려움을 해결하기 위해 사용되었던 방법들은 (복수 답변 가능)?

A. 피험자가 직접 병원 방문하는 대신 전화로 방문

B. 전자 동의서 사용

C. 원격으로 데이터 수집

D. 병원 방문 대신에 피험자 집 근처 의료기관 방문하여 검사 진행

E. 병원에 방문하여 임상시험용의약품 받아 가는 것 대신에 택배를 이용하여 의약품 배달

F. 피험자가 병원 방문하는 대신에 가정간호사가 피험자 집을 방문

G. 스폰서 직원이 병원 방문하는 대신에 전화나 이메일로 연락을 주고 받음

11. 코로나19가 종식되면 임상시험 운영 방법에 어떤 변화가 있을지요?

A. 과거 운영 방법으로 돌아가 변화가 없을 것이다.

B. 과거와는 다르게 원격 진료 방법이나 의약품 택배 등 다양한 새로운 방법으로 진행될 것이다.

12. 11번에 과거와 다르게 새로운 시도가 될 것으로 답변 하신 경우, 어떤 것들이 시도될 것으로 예측하시는지요 (복수 답변 가능)?

A. 전자동의

B. 원격진료

C. 피험자 집으로 임상시험 의약품 배송

D. 전자 기기를 이용한 임상시험관련 정보를 원격으로 수집

E. 가정간호사를 이용하여 피험자 집을 방문

F. 일부 검사는 병원에 오지 않고 피험자 집 근처 의원을 방문하여 실시

G. 스폰서 CRA가 병원에 방문하여 모니터링하지 않고 원격으로 EMR 접속하여 remote monitoring

13. 원격진료 등 새로운 시도를 이용하는 임상시험을 부르는 용어로 다음 중 어떤 것을 들어 보셨나요?

A. 가상임상시험 (Virtual clinical trial)

B. Decentralized clinical trial

C. Remote clinical trial

D. Direct to Patient Trial

14. 새로운 임상시험 시도에 대해서 제안하거나 도움이 필요한 것들에는 무엇이 있을지요? 보건당국, 의뢰사, 병원 등에 대하여 구체적으로 요청 사항을 기술해 주시기 바랍니다.

A. 보건 당국:

B. 의뢰사:

C. 병원:

D. 기타 요청 사항:

설문에 답변하여 주셔서 감사 드립니다.

설문 결과는 [redacted] 에게 보내 주시기 바랍니다.
